# Prediction of melanoma metastasis by the Shields index based on lymphatic vessel density

**DOI:** 10.1186/1471-2407-10-208

**Published:** 2010-05-17

**Authors:** Maxine S Emmett, Kirsty E Symonds, Howard Rigby, Martin G Cook, Rebecca Price, Chris Metcalfe, Antonio Orlando, David O Bates

**Affiliations:** 1 Microvascular Research Laboratories, Bristol Heart Institute, Department of Physiology and Pharmacology, University of Bristol, Bristol, UK; 2 Department of Pathology, Frenchay Hospital, Bristol. UK; 3 Royal Surrey County Hospital and University of Surrey, Guildford, UK; 4 Department of Social Medicine, University of Bristol, Bristol, UK; 5 Department of Plastic Surgery Frenchay Hospital, Bristol, UK

## Abstract

**Background:**

Melanoma usually presents as an initial skin lesion without evidence of metastasis. A significant proportion of patients develop subsequent local, regional or distant metastasis, sometimes many years after the initial lesion was removed. The current most effective staging method to identify early regional metastasis is sentinel lymph node biopsy (SLNB), which is invasive, not without morbidity and, while improving staging, may not improve overall survival. Lymphatic density, Breslow's thickness and the presence or absence of lymphatic invasion combined has been proposed to be a prognostic index of metastasis, by Shields et al in a patient group.

**Methods:**

Here we undertook a retrospective analysis of 102 malignant melanomas from patients with more than five years follow-up to evaluate the Shields' index and compare with existing indicators.

**Results:**

The Shields' index accurately predicted outcome in 90% of patients with metastases and 84% without metastases. For these, the Shields index was more predictive than thickness or lymphatic density. Alternate lymphatic measurement (hot spot analysis) was also effective when combined into the Shields index in a cohort of 24 patients.

**Conclusions:**

These results show the Shields index, a non-invasive analysis based on immunohistochemistry of lymphatics surrounding primary lesions that can accurately predict outcome, is a simple, useful prognostic tool in malignant melanoma.

## Background

Melanoma, the most lethal form of skin cancer can be highly metastatic. The most common site of metastatic disease in melanoma is the regional lymph nodes indicating that metastatic spread usually occurs via the lymphatic system. Regional lymph node metastasis is associated with a poor prognosis, with 10-year survival rates of 35%[1]. The most widely used prognostic indicator for survival is Breslow thickness, however, this is still inaccurate for a significant number of patients[1]. A significant proportion (15%) of patients with invasive thin tumours (<1 mm), predicted to be low risk for spread, still develop metastatic disease. There are currently no accepted prognostic indicators to determine which of these patients with thin melanoma will develop metastasis. Similarly, a substantial proportion of patients with thick melanoma will have long term survival (45% greater than 10 years) and not develop regional or distant spread.

Under current clinical practice patients with confirmed melanoma are staged according to Breslow thickness and Clark's levels and in most centres are offered sentinel lymph node biopsy (SLNB) (particularly if tumours are thicker than 2 mm)[2]. After excision of the primary melanoma, if the patient is node negative on clinical examination, or by SLNB, they are routinely followed up every three months for five years. The sentinel lymph node (SLN) is the first node draining the area of skin around the primary malignant melanoma and is excised for histological analysis. If the node is found to be positive (SLN+ve) then regional lymph node clearance is usually carried out, whereas patients who are SLNB negative (SLN-ve) are followed up as usual, every three months for up to five years. SLN+ve patients do not appear to benefit in terms of overall survival from the subsequent lymph node clearance surgery and there is no strong evidence to show that it results in improvement in the rate of recurrence or spread of the melanoma[2]. SLN-ve patients on the other hand have a 15% 5-year mortality, compared with 53% mortality for SLN+ve patients[3]. It is clear therefore that SLNB is a useful indicator of likelihood of recurrence[4], although it is has been proposed that it is insensitive for 18-22% of patients (incorrectly predicts as disease free)[5], and non-specific for 47% of patients (incorrectly identifies them as likely to die from recurrence) if 5-year survival statistics are used as an endpoint[3]. In addition it requires significant theatre time, and can be a cause of morbidity in a proportion of patients, so there is still discussion in the literature of its value and cost effectiveness[6]. It is thought that SLNB predicts melanoma metastasis as melanoma cells that gain access to the lymphatic system surrounding the primary lesion subsequently drain into the sentinel lymph node, being seen as metastases, which can then seed subsequent metastases to more distant lymph nodes[7].

Despite the uncertainty surrounding the mechanisms underlying metastasis, there is a clear difference in lymphatic vessel density (LVD) surrounding primary malignant melanomas, which then develop metastases, compared with non-metastatic malignant melanomas [8,9]. As a result, lymphatic density (number of lymphatics per square mm bordering the melanoma) has been suggested as a prognostic indicator for the progression and spread of malignant melanoma[8-11]. This was extended by Shields *et al*. (2004) to develop a prognostic index in a small cohort of patients. This index considered tumour thickness, peri-tumoral LVD and lymphatic invasion, all of which contribute to metastasis, to generate a more efficient predictor. The index was weighted to bias factors that showed the greatest correlation with metastasis. Therefore lymphatic vessel density was squared, as it appeared to be the most important prognostic factor, whilst lymphatic invasion was given a value of 2 if malignant cells were seen to be present within LYVE-1 positive vessels, and a value of 1 if none were found. Using this Shields Index it was possible to differentiate tumours that subsequently metastasised from those that did not, in a small cohort of 21 patients, limiting its clinical value. From such a small sampling it provides only limited analysis with regards to how robust the test is and the weighting and value of each factor considered. In order to consider the Shields Index for the clinical setting these assessments are necessary. Our objective was to determine whether the Shields index is an effective predictor of metastatic status in patients that were clinically metastasis free upon excision of the primary melanoma. We also set out to compare current predictive methodology with the Shields index to determine the best method of predicting metastatic outcome for patients.

## Methods

The study conforms to the guidelines for Reporting Recommendations for Tumor Marker Prognostic Studies (REMARK)[12]. Specific components of the REMARK guidelines are identified in the text by numbers in diamond brackets.

### Patient details

The Frenchay Hospital Melanoma Registry contains original samples taken at time of excision, information on Breslow thickness, and metastatic outcome (i.e. whether or not the patient went on to develop metastases), followed up for a period of at least ten years on patients treated by excision of melanomas from 1979. Melanoma tissues were randomly selected from 102 patients from the Registry, excluding any tumours that are only *in situ *and then selecting participants under the following criteria: clear of metastases at time of excision, with Breslow thickness < 8 mm, with > 5-years of follow-up, and with no signs of ulceration 〈1,2〉, with ethical approval from North Bristol Research Ethics Committee (H7/0102/45). Sections of melanomas, excised by wide local excision, fixed in paraformaldehyde, cut into ~2 mm thick samples, and paraffin embedded were used to calculate Breslow thickness〈3〉. Samples were stored as paraffin embedded blocks〈4〉. 57 patients did not develop clinically apparent metastases within this follow-up period, described as non-metastatic, 45 patients later developed either regional or distant metastasis arising from the primary tumour, described as metastatic〈7〉. 49 additional patient samples were selected but not used for analysis. (5 had a thickness > 8 mm, eight had indistinct borders of the melanoma so LVD could not be calculated, 16 could not stain with LYVE-1, five were lost due to poor preservation, four were subsequently assessed to be melanoma in situ, and eleven were lost to follow up 〈10〉.

18 additional patients had undergone SLNB at the Royal Surrey Hospital, London. 8 were negative for signs of metastasis at time of biopsy whereas 10 were positive. All patients were negative for signs of metastasis at time of primary tumour excision and were matched for Breslow thickness.

### Immunohistochemistry

Paraffin embedded sections were dewaxed re-hydrated, and microwave antigen retrieval carried out in 0.01 M sodium citrate, pH 6.0, 10 minutes at 800 W incubated in 3% hydrogen peroxide for 5 minutes, washed twice in phosphate buffered saline (PBS (mM): NaCl, 137; KCl, 2.68; Na2HPO4, 10; KH2PO4, 1.76), incubated in 1.5% normal horse serum in 1 × PBS for 30 minutes, and overnight at 4°C in a humid chamber with polyclonal goat anti-human LYVE-1 antibody; 15 μg/ml (AF2089, R&D Systems, UK) or normal goat IgG (1-5000, Vector Laboratories, Peterborough, UK), in non-immune block (Zymed Laboratories, San Fransisco, USA). Slides were washed twice in PBS/Tween, 0.05% v/v, (PBT) before blocking and incubation with biotinylated horse anti-goat IgG secondary antibody (2 μg/ml BA-9500, Vector Laboratories, Peterborough, UK) in blocking solution, in a humid chamber for 30 minutes. Sections were washed twice with 1 × PBT for 5 minutes before incubating for 30 minutes with Elite ABC Kit, (Vector Laboratories, Peterborough, UK) at room temperature. Sections were visualised with diaminobenzidine (DAB, Vector Laboratories, Peterborough, UK) and washed in distilled water, haematoxylin counterstained and DPX mounted.

### Lymphatic vessel density

Sections were analysed independently by three experienced researchers (DOB, KES and MSE). The researchers were blinded to the status and reported Breslow thickness of the melanoma, and the patients were anonymised to the researchers. There was a high degree of reproducibility between the observers with >90% of samples having the same number of lymphatics identified to within 2. Lymphatic vessels were identified under a Nikon E400 microscope as structures positive for LYVE-1 staining. Total epi-tumoural lymphatic vessel density (LVD) was calculated using a × 40 objective, counting every lymphatic within a 350 μm border around the tumour edge. Digital images were taken to form a composite image and epi-tumoural area calculated using NIH Image J. Hot spot analysis (identification of areas of subjectively determined high lymphatic density) of epi-tumoral LVD from 24 patients was assessed using a × 100 objective as previously described[10].

### Shields Index 〈5〉

The Shields Index[8,9] (Box 1) was calculated from LVD, Breslow thickness (thickness) and the presence or absence of lymphatic vessel invasion (LVI). Lymphatic vessel invasion was assessed using a × 40 objective and defined as the presence of tumour cells within a LYVE-1 positive vessel within the primary tumour〈11〉.

Calculation of the Shields Index

### Shields Index = (LVD^2 ^× invasion) × (Breslow thickness) 〈1〉

LVD is the Total peri-tumoural lymphatic vessel density

Invasion = 2 if lymphatic invasion present, 1 otherwise

### Statistical analysis〈10〉

AJCC stage, LVD, Breslow thickness, incidence of lymphatic invasion, and the Shields index were considered as prognostic variables 〈8〉. Logistic regression models were used to estimate associations between each of these variables in turn and subsequent occurrence of metastasis. The discriminatory power of each variable was examined using receiver operator characteristic (ROC) curves, and areas under those curves (AUC). AUCs can be considered the probability that the variable is at a higher level for case compared with control, and can be compared between variables using the test described by Hanley[13]. The sample size of 45 patients with metastases was anticipated to allow a sensitivity of 90% to be estimated with 95% confidence interval of 76% to 96% 〈9〉.

There was no significant difference between metastatic and non-metastatic patient groups in mean Breslow thickness, patient age, or Clarks Level (Table 1) 〈13〉. However, mean follow up time (time since excision of primary tumour) was significantly longer in the non metastatic group, compared with metastatic group 〈6〉.

**Table 1:** Comparison of the metastatic and non-metastatic patient groups.

	Age (years)	Breslow Thickness (mm)	Time Since Excision of Primary Lesion (months)	Clarks Level
Metastatic	57.23 ± 17.4	2.4 ± 1.6	53 ± 36	4.06 ± 0.26
Non-metastatic	55.63 ± 16.4	1.911 ± 1.4	92 ± 36	4.05 ± 0.37

There was no significant difference between metastatic and non-metastatic patient groups, in patient age (p > 0.05, one-way ANOVA, Bonferroni), Breslow thickness (p = 0.17 Mann Whitney U test), or Clarks level (p > 0.05, one-way ANOVA, Bonferroni). Mean follow up time (time since excision of primary tumour) was significantly longer in the non-metastatic group, compared with metastatic group (p < 0.0001 unpaired t test). All results represent mean ± s.d.

Logistic regression models were used to estimate associations between metastasis and each of the following: Breslow thickness, LVD, LVI and the Shields Index, The prognostic power of each test was assessed using Receiver Operator Characteristic Curves (ROC Curves) and the area under the curve compared as described by Hanley[13].

## Results

### Epi-tumoural lymphatic density and the shields index

**Figure 1:**
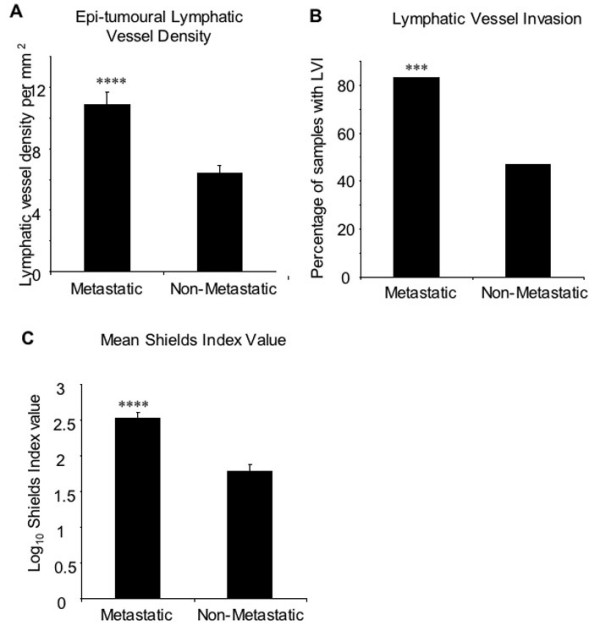
**Mean epi-tumoural lymphatic vessel density and lymphatic vessel invasion. **Lymphatic vessel density was significantly higher in tumours from patients that subsequently developed metastases (metastatic), compared to non-metastatic tumours (p < 0.0001, unpaired t test). B) There were a greater percentage of tumours with signs of LVI in the metastatic patient group compared to the non-metastatic group (p < 0.001 Fisher's Exact Test). C) Shields Index values were calculated by combining peritumoural LD, LVI and Breslow thickness. Mean Shields Index value was significantly higher in patients that subsequently developed metastases (metastatic), compared to non-metastatic tumours (p < 0.0001, unpaired t test). Error bars represent standard error of the mean

Of the 102 patients selected from the Frenchay Hospital Melanoma Registry, 45 were cases having developed metastases within five years of follow-up, and 57 were controls. There was strong evidence that metastasis was associated with a higher mean LVD (Table 2, Figure 1A, p = 0.001), a higher risk of lymphatic vessel invasion (Table 2, Figure 1B, p < 0.001), but no convincing evidence of an association with Breslow thickness (Table 2, p = 0.15). With regards to LVD, the mean densities in both cases and controls are within the range of densities found in normal dermis, which was described by Joory *et al*. as falling between 0 to 25.1 mm^-2 ^with a skewed right Poisson-like distribution with a mean 10.6 ± 0.67 mm^-2 ^[9,14]. Similarly there was strong evidence of an association between the Shields Index and subsequent metastasis within five years (Table 2, Figure 1C, p < 0.001). For each one log10 unit increase in the Shields index, there was an almost ten-fold increase in the odds of metastases 〈7,15〉.

**Table 2:** Associations between the Shields Index, the component measures, and subsequent metastases.

	Metastasis at 5 yrs (n = 45)		No metastasis at 5 yrs (n = 57)		Odds ratio	(95% CI)	p
Mean lymphatic vessel density, vessels/mm^2 ^(SD)	10.24	(5.54)	6.52	(3.82)	1.19	(1.08, 1.31)	0.001
Number with lymphatic vessel invasion (%)	38	(84)	26	(46)	6.47	(2.48, 16.90)	<0.001
Mean Breslow thickness, mm (sd)	2.36	(1.55)	1.93	(1.37)	1.23	(0.93, 1.61)	0.15
Log shields index (sd)	2.43	(0.50)	1.77	(0.62)	9.86	(3.57, 27.24)	<0.001

The odds ratios are for a one unit increase in LVD, Breslow thickness and the log_10 _Shields Index, and for the comparison between patients with and without lymphatic vessel invasion.

### Discriminatory power of the different measures

**Figure 2:**
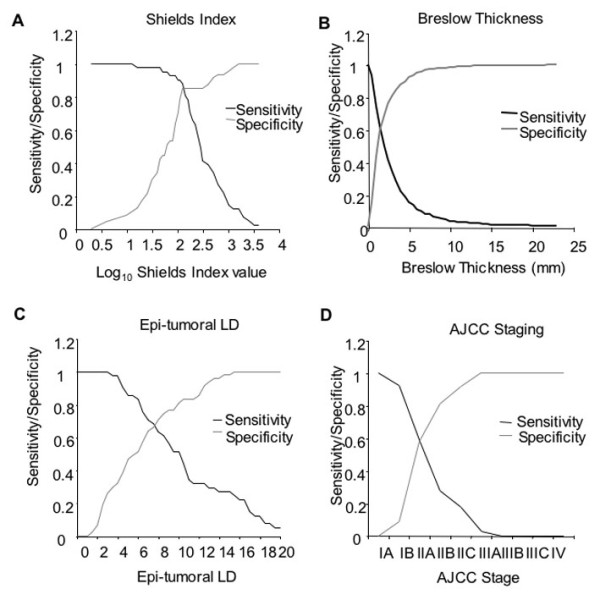
**Sensitivity and specificity curves for the different prognostic factors. **The sensitivity (i.e. the number of correctly predicted patients with metastatic spread) and specificity (i.e. the number of correctly predicted patients that did not develop metastases) were plotted on the same axes as a representation of the predictive value of the prognostic test. The point at which the two lines intersect represents the optimal cut-off point for determining a positive or negative test result in order to achieve the best sensitivity and specificity for the test. A) Shields Index B) Breslow thickness for the 4500 patients included in the Frenchay Hospital Melanoma Registry C) Epi-tumoural LD D) AJCC stage.

**Figure 3:**
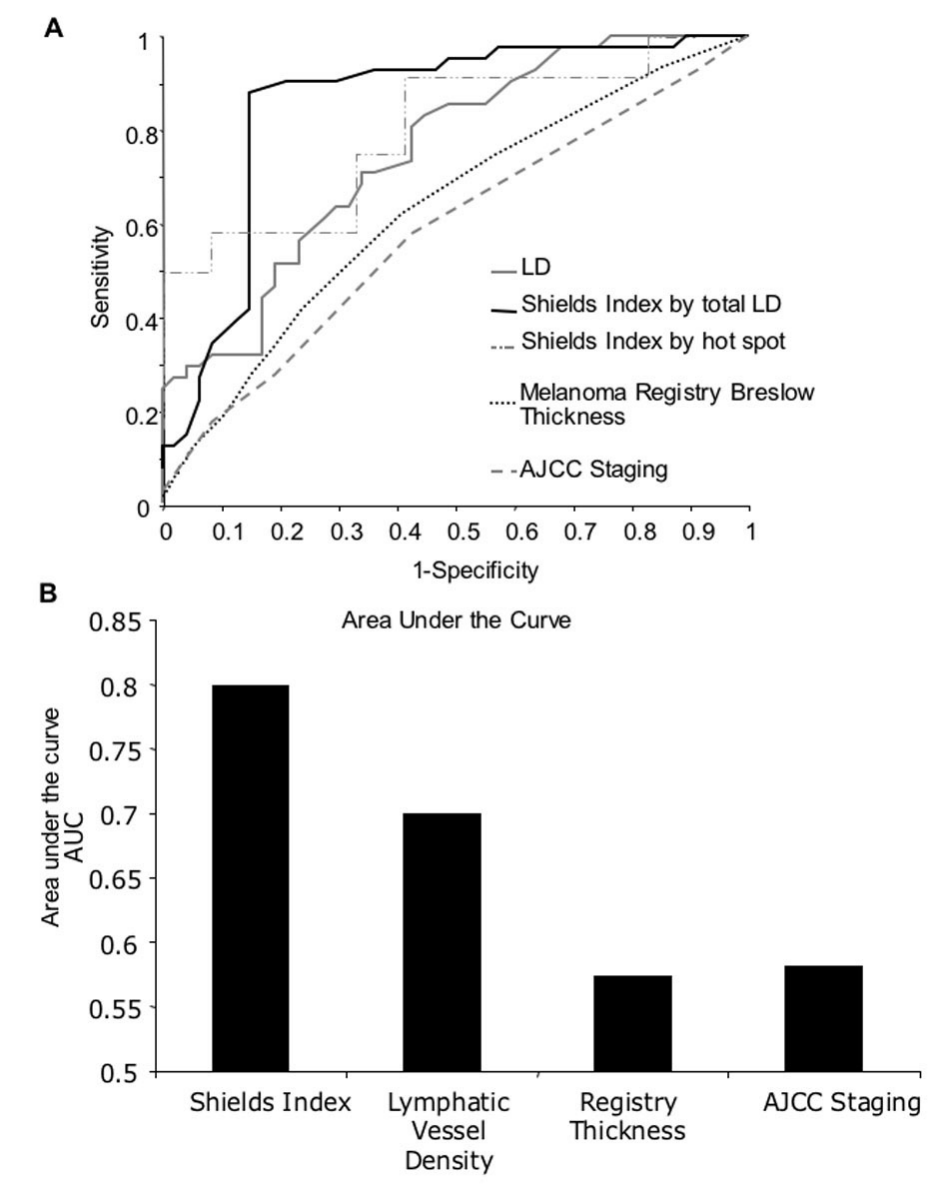
**Receiver Operator Characteristic curves. **A. Receiver Operator Characteristic (ROC) curves were generated by plotting the sensitivity (i.e. the proportion of times the test correctly predicts a patient as metastatic) against 1-specificity (i.e. 1-the proportion of times the test correctly predicts a patient as non-metastatic) as a measure of the predictive value for each test. Shields Index calculated by total LD and by hot spot, LD, Breslow thickness, Breslow thickness for the 4500 patients included in the Frenchay Hospital Melanoma Registry and AJCC staging. B. The area under the ROC curve (AUC) represents numerically the accuracy of the test. A prognostic indicator with 100% sensitivity and 100% specificity has an AUC of 1.0. The most accurate prognostic indicator is the Shields Index, followed by epi-tumoural LD, Breslow thickness and finally AJCC staging

Sensitivity specificity curves (figure 2), ROC curves and AUCs indicate that the Shields Index achieves the greatest discriminatory power, with AJCC staging performing least well (Figure 3) in this group of patients who were without lymph node or other metastasis at the time of melanoma excision. The Shields Index achieved an AUC of 0.82 (95% CI 0.73 to 0.90), significantly better than the AUC of 0.70 for lymphatic vessel density (95% CI 0.60 to 0.80, p for comparison = 0.002). AJCC staging achieved an AUC of 0.58 in this cohort while Breslow thickness achieved an AUC of 0.58 in the 4,500 patients listed in the Frenchay Hospital Melanoma Registry.

**Figure 4:**
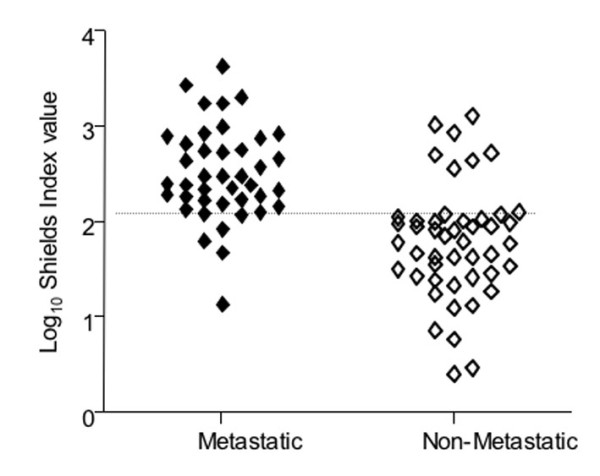
**Shields Index values scatterplot. **The Shields Index values, for all 106 patients assessed, were plotted on a scatterplot to represent the spread in Shields Index values for the metastatic and non-metastatic groups. The Shields Index (calculated from epi-tumoural LD, lymphatic vessel invasion and Breslow thickness). With a cut off point of 2.10, 92 of the 106 samples are correctly predicted (86.8%) with only 14 samples predicting incorrectly. A total of 5 patients that later developed metastases were predicted to be non-metastatic and 9 patients that have not yet developed metastases are predicted to do so.

Adopting a cut-off of 2.1 for the Shields Index (Figure 4), 37 out of 45 patients with metastases within 5 years exceed the cut-off, a sensitivity of 82%. Of the 57 patients who did not develop metastases within five years, 46 fell below the cut-off, a specificity of 81%. 〈16,17〉.

### Sentinel Lymph Node Biopsy

**Figure 5:**
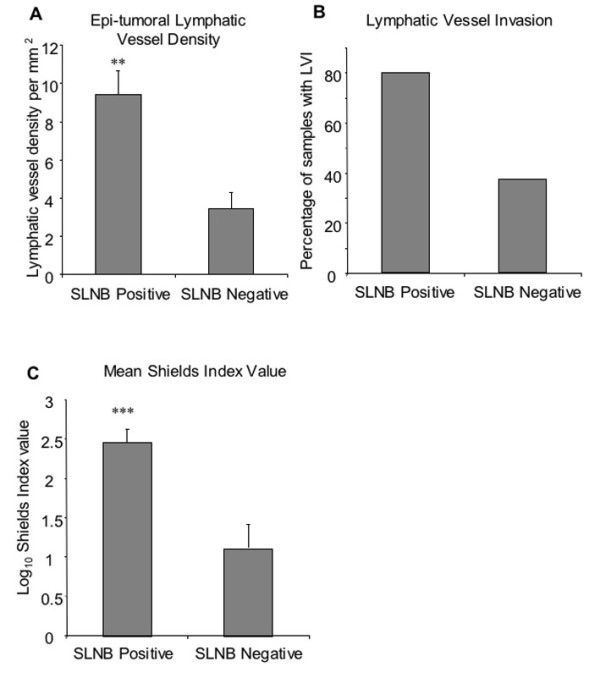
**Comparison of epi-tumoural lymphatic vessel density, lymphovascular invasion, shields index and sentinel lymph node biopsy. **Shields Index values were calculated on primary tumours from SNLB patients A) Mean epi-tumoural LD (p < 0.01, unpaired t test). B) Percentage of patients that were positive for LVI (p > 0.05, Fisher's Exact Test). C) Mean Shields Index values (p < 0.001, unpaired t test) for SLNB positive and negative patients. Error bars represent standard error of the mean.

In the 18 patients that had previously undergone SLNB, epi-tumoural LVD was significantly higher in patients who were positive for signs of SLN metastasis (8.2 ± 1.1 mm^-2^) compared to patients that were negative, (4.9 ± 1.8, Figure 5A, p < 0.01, unpaired t-test). Lymphatic vessel invasion was also increased in SLNB positive patients, with 80% of SLNB positive patients positive for invasion compared to only 37.5% of SLNB negative patients (Figure 5B, p = 0.145, Fisher's Exact Test). The mean Shields Index value was significantly higher in SLNB positive patients compared to negative patients (Figure 5C, p < 0.001, unpaired t test).

### Hot Spot Analysis

**Figure 6:**
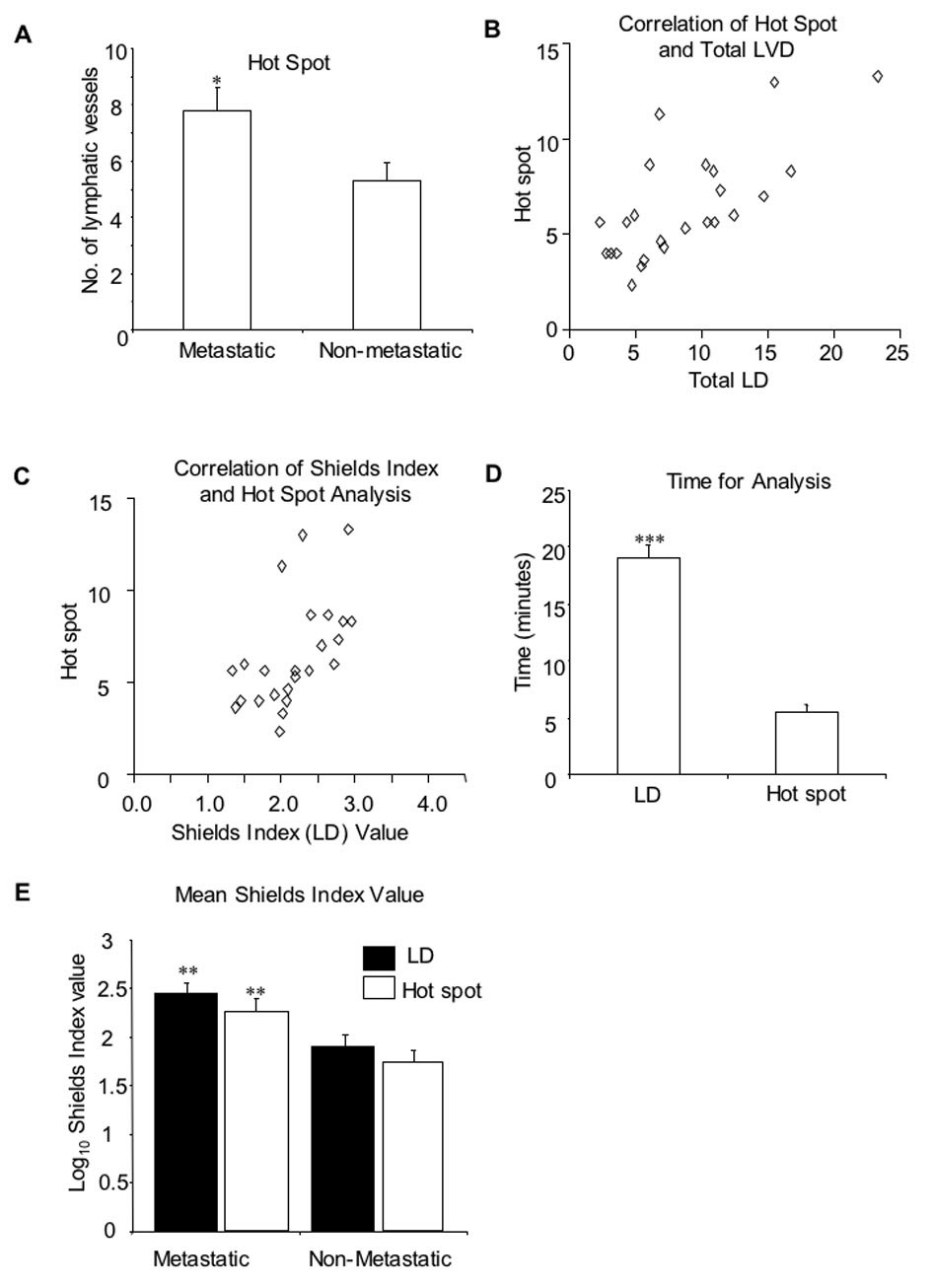
**Comparison of total lymphatic vessel density and hot spot analysis. **Hot spot analysis and total LD were calculated. Hot spot analysis assessed lymphatic number by counting the number of immunohistochemically stained lymphatic vessels, in three regions with the highest LD, as assessed by eye, in a 100 μm border around the tumour. A) Hot spots (p < 0.05, unpaired t test) in 12 patients that later developed metastases (metastatic) and 12 patients which after 9 years follow-up did not. Error bars represent standard error of the mean. B) Total epi-tumoural LD and hot spot analysis were significantly correlated (p < 0.0001, Pearson, *r^2 ^*= 0.50). C) The Shields Index and hot spot analysis significantly correlated together (p < 0.01, Pearson, *r^2 ^*= 0.30) D) Time taken to calculate a Shields Index value compared to the time taken to calculate hot spot analysis in the same subset of patients (p < 0.0001, unpaired t test). E) Shields Index calculated by LD or hotspot are not different from each other.

A subset of 24 patients from the Frenchay Melanoma Registry, were assessed for epi-tumoural LVD by both total and hot spot analysis. Similar to total epi-tumoural LVD assessment, hot spot analysis demonstrated an increase in the number of lymphatics around metastatic compared to non-metastatic tumours (Figure 6A, 7.75 vessels/mm^2^, metastatic, 5.28 vessels/mm^2^, non-metastatic, p < 0.05, unpaired t-test). There was a high level of correlation between both the total epi-tumoural LVD and hot spot analysis (Figure 6B, p < 0.0001, Pearson, r^2 ^= 0.50) and Shields Index and hot spot analysis (Figure 6C, p < 0.01, Pearson, r^2 ^= 0.30). The time taken to undertake both the Shields Index and hot spot analysis were also compared, with the Shields Index taking an average of four times longer than hot spot analysis (Figure 6D, 19.0 ± 1.1 minutes, Shields Index, 5.5 ± 0.6 minutes, hot spot analysis, p < 0.0001, paired t-test. When the Shields Index was calculated using total epi-tumoural LVD in this small subset of patients, mean Shields Index for metastatic patients was 2.5 ± 0.1 compared to 1.9 ± 0.1 for non-metastatic patients (Figure 6E, p < 0.01, unpaired t-test). When the Shields Index was calculated in this same patient cohort using hot spot analysis, there was still a significant difference between the two groups, with a mean Shields Index value of 2.3 ± 0.1 for metastatic patients compared to 1.7 ± 0.1 for non-metastatic patients (Figure 6E, p < 0.01, unpaired t-test). There was no statistical significant difference between mean Shields index calculated using the two methods (hot spot or LVD). The Shields index calculated by hot spot analysis gave a lower prognostic power than by LVD (AUC 0.75, p < 0.05 compared with LVD Shields index).

## Discussion

There is still some controversy over the management of melanoma patients[6,7]. Sentinel lymph node biopsy is used as a staging method for identification of non palpable regional lymph node metastasis [2,7]. However while a positive lymph node finding may alter management of the condition it does not improve overall survival of the patient[2]. SNLB is costly, and invasive with >10% of patients experiencing significant morbidities[4]. Yet it is clearly more accurate than other prognostic factors for patients node negative on clinical assessment, particularly for those with intermediate thicknesses of melanoma. SLNB can clearly aid staging, trial choice, etc. It does however, have a significant insensitivity (15-20%), and a substantial non-specificity (45%), meaning that many patients are put at unnecessary concern that they have metastatic disease, and some have metastases that are missed. The results we describe here provide a complementary system to aid in prognostic determination of melanoma. The Shields index is more specific and at least equally sensitive compared to SLNB, and is accurate outside the safety net of SLNB patients. In the group described here the Shields index was accurately able to predict both metastasis derived from melanomas <2 mm, and a lack of metastasis in thick melanomas (>4 mm). 〈19〉

There have been two primary methods for determining lymphatic vessel density surrounding tumours. The method primarily used here is an objective lymphatic density counted from cross sections of LYVE-1 stained lymphatic vessels per mm^2 ^of section in a 350 μm (one high powered field) border around the tumour. The alternative method involves counting vessels within three hot spots of lymphatics surrounding the tumours[10]. There was little difference in the accuracy of the methodology when directly compared here, and although the hot spot method had a slightly lower area under the curve value, and was subjective, it was quicker. The Shields index appears to be valid independent of the method of LVD assessment. Although there is now an array of lymphatic specific markers available, lymphatics were examined herein through the sole use of LYVE-1. LYVE-1 is highly specific and has been shown to be expressed almost exclusively on the lymphatic endothelium [15]. Furthermore, direct comparisons of lymphatic vessel density calculated with either LYVE-1 or other lymphatic markers, has demonstrated that LYVE-1 offers the most accurate assessment (unpublished data).

It is now clear that in animal models the incidence of metastasis is increased in tumours expressing lymphatic endothelial growth factors such as vascular endothelial growth factor C (VEGF-C) [16]. Moreover, it has been shown that areas of concentrated lymphatic endothelial cell populations can stimulate the migration of melanoma cells towards them resulting in melanoma growth to areas of high lymphatic density through either directed metastatic chemotaxis [17], and/or autologous chemotaxis [18]. This has led to the hypothesis that tumours that stimulate lymphangiogenesis, or can recognise areas of high lymphatic density, will be more likely to metastasise than tumours that do not.

The two mechanisms underlying increased lymphatic density - chemotaxis and lymphangiogenesis - are also now being understood at the molecular levels. Increased VEGF-C production by melanomas can not only induce lymphangiogenesis, but also increase lymph flow, thus aiding fluid drainage from the tumour. This fluid drainage can serve to increase metastasis both by passively carrying tumour cells and by establishing autocrine chemotactic gradients by autologously secreted heparin binding growth factors such as VEGF-A or CCL21. Furthermore, VEGF-C secreted by the tumour can result in lymph node lymphangiogenesis [19], which results in a more permissive environment for metastases from micrometastases [20]. The second mechanism underlying increased lymphatic density is metastatic chemotaxis. Tumour cells that upregulate chemokine receptors for molecules secreted by lymphatic endothelial cells recognise areas of lymphatic endothelial cell concentrations and grow towards areas of high lymphatic density, or hot spots[17,21]. These chemokines, such as CCL21 are used by receptor expressing tumour cells (CCR7 for instance is upregulated in metastatic but not non metastatic melanoma cells[22]) and provide a route out of the primary melanoma to the lymph node. Thus increased lymphatic density predicts the likelihood of SLNB positivity. It will be interesting to determine whether combining SLNB positivity with a high Shields value will increase the already high sensitivity of each test, and whether a low Shields value can be used to reduce SLNB in patients at low risk.

## Conclusions

We show that the use of the index described by Shields *et al*. in 2004 is the most specific and sensitive method to predict metastatic outcome (i.e. whether or not the patient went on to develop metastases) in this group of 102 melanoma patients. This method is cheap, reproducible, and provides no additional invasive procedures from the original primary excision of the tumour. These findings suggest that selection of patients for SLNB could be informed by the Shields' index values. Thus SLNB could be targeted more towards those patients with thin melanomas but high Shields index. It also suggests that staging of metastatic melanoma should include this parameter, so refining Breslow thickness [20].

## Competing interests

The authors declare that they have no competing interests.

## Authors' contributions

MSE and KES did the LYVE-1 staining and vessel counting and analysed the data, MSE and HR selected the patient samples and confirmed the pathology, MGC provided pathology expertise on the SLN biopsy samples, RP identified metastatic samples, CM conceived and assisted with the statistical analysis, AO and DOB conceived the study, supervised, gained the funding and with MSE wrote the paper.

## Pre-publication history

The pre-publication history for this paper can be accessed here:

http://www.biomedcentral.com/1471-2407/10/208/prepub
